# MicroRNA-200b Regulates the Proliferation and Differentiation of Ovine Preadipocytes by Targeting *p27* and *KLF9*

**DOI:** 10.3390/ani11082417

**Published:** 2021-08-17

**Authors:** Xiayang Jin, Jiqing Wang, Jiang Hu, Xiu Liu, Shaobin Li, Yujie Lu, Huimin Zhen, Mingna Li, Zhidong Zhao, Yuzhu Luo

**Affiliations:** Gansu Key Laboratory of Herbivorous Animal Biotechnology, Faculty of Animal Science and Technology, Gansu Agricultural University, Lanzhou 730070, China; jinxiayang2018@163.com (X.J.); huj@gsau.edu.cn (J.H.); liuxiu@gsau.edu.cn (X.L.); lisb@gsau.edu.cn (S.L.); luyj@st.gsau.edu.cn (Y.L.); zhenhm@st.gsau.edu.cn (H.Z.); limn@gsau.edu.cn (M.L.); zhaozd@gsau.edu.cn (Z.Z.)

**Keywords:** miR-200b, proliferation, differentiation, ovine preadipocytes, *KLF9*, *p27*

## Abstract

**Simple Summary:**

The miR-200b has been shown to play an important role in preadipocyte proliferation and differentiation. Herein, we explored the role of miR-200b in ovine adipocyte development, using Oil Red O staining, cell viability analysis, EdU and RT-qPCR. The results showed that miR-200b facilitated proliferation and suppressed the differentiation of preadipocytes. The dual fluorescent reporter vector experiments showed that miR-200b directly targeted *p27* and *KLF9*. Meanwhile, we demonstrated that *p27* significantly inhibited the proliferation, while *KLF9* significantly promoted the differentiation of preadipocytes.

**Abstract:**

MicroRNAs (miRNAs) are crucial regulatory molecules in lipid deposition and metabolism. However, the effect of miR-200b on the regulation of proliferation and adipogenesis of ovine preadipocytes is unknown in the sheep (*Ovis aries*). In this study, the expression profiles of miR-200b were investigated in the seven tissues of Tibetan ewes and differentiated preadipocytes. The effect of miR-200b, as well as its target genes *p27* and *KLF9*, on the proliferation of ovine preadipocytes and adipogenesis was also investigated, using cell viability analysis, EdU staining, Oil Red O staining and reverse transcription-quantitative PCR (RT-qRCR). The miR-200b was expressed in all the tissues investigated, and it was highly expressed in lung, liver, subcutaneous adipose and spleen tissues. The expression of miR-200b continuously decreased when the differentiation of ovine preadipocytes initiated. The miR-200b mimic dramatically accelerated the proliferation but inhibited differentiation of ovine preadipocytes. The miR-200b inhibitor resulted in an opposite effect on the proliferation and differentiation of ovine preadipocytes. The dual luciferase reporter assay results showed that miR-200b mimic significantly decreased the luciferase activity of *p27* and *KLF9* in HEK293 cells transfected with wild-type dual luciferase reporter vectors. This suggests that *p27* and *KLF9* are the target genes of miR-200b. In over-expressed-*p27* preadipocytes, the number of EdU-labeled preadipocytes and the expression levels of proliferation marker genes *CDK2*, *CDK4*, *CCND1* and *PCNA* significantly decreased. In addition, the transfection of over-expressed-*KLF9* vector into adipocytes remarkably increased the accumulation of lipid droplets and the expression levels of differentiation marker genes *aP2*, *PPARγ*, *LPL* and *GLUT4*. These results suggest that miR-200b accelerated the proliferation but inhibited the adipogenic differentiation of ovine preadipocytes by targeting *p27* and *KLF9*, respectively.

## 1. Introduction

The growth of adipose tissue is one of important factors affecting meat production performance and meat quality of domestic animals, especially for the tenderness and flavor of meat [1,2]. Preadipocytes are a class of cells widely distributed in adipose tissue, while their proliferation and differentiation are responsible for growth and development of adipose tissue in mammals [3]. It has been shown that the activities of preadipocytes are complex and precisely orchestrated processes and many regulatory factors are involved in the activities [4,5]. In this context, elucidating the molecular mechanisms regulating preadipocyte proliferation and differentiation is of academic and economic importance for improving meat quality in sheep. It has been known from previous studies that microRNAs (miRNAs) are involved in the regulation of the proliferation and differentiation of preadipocytes [6,7].

The miRNAs are evolutionarily conserved non-coding RNAs with approximately 21–25 nucleotides in length. They can either promote degradation or repress translation of the target mRNAs [8]. There are some studies that have demonstrated the regulation roles of miRNAs in the proliferation and adipogenic differentiation [9]. For example, the over-expression of miR-125a-5p promoted the proliferation of porcine preadipocytes. On the contrary, the inhibition of the miRNA prevented the differentiation of preadipocytes [10]. The upregulated miR-193b was found to promote the proliferation of bovine preadipocytes, but inhibited its differentiation [11]. The function of other miRNAs in the proliferation and differentiation of preadipocytes has also been reported, including miR-330-5p in sheep [12], miR-204 in cattle [13], and miR-181a [14], miR-146a-5p [15], and miR-196a in pigs [16].

The miR-200 family consists of miR-200a, miR-200b, miR-200c, miR-429, and miR-141. The sequences of the five members are highly conserved within their seed sequence. However, to date, the functional studies of miR-200b have mainly focused on tumor cells [17,18]; there have been few reports in the adipogenesis of preadipocytes. In the 3T3-L1 preadipocytes, miR-200b has been reported to inhibit the adipogenesis by downregulating the expression level of Krüppel-like factor 4 (*KLF4*) [17]. In transgenic mouse models, the knockout of miR-200b resulted in high fat diet induced obesity and insulin resistance [19]. These suggest that miR-200b can inhibit the differentiation of preadipocytes and the deposition of adipose tissues in mice. However, to our knowledge, there have been no reports on the function of miR-200b in the regulation of proliferation and differentiation of preadipocytes, and lipid deposition in the sheep.

In this study, we investigate the expression characterization of miR-200b and its effects on the proliferation and differentiation of ovine preadipocytes. We also verify the target relationship between miR-200b and cyclin-dependent kinase inhibitor 1 B (*p27*) and Krüppel-like factor 9 (*KLF9*), and finally evaluate the regulatory effect of miR-200b on the expression of these target genes, and the effect of *p27* and *KLF9* on the proliferation and differentiation of ovine preadipocytes.

## 2. Materials and Methods

### 2.1. Ethics Statement

The animal work was carried out in accordance with the rules for the care and use of laboratory animals published by the Ministry of Science and Technology of the People’s Republic of China (Approval number 2006–398) and was also approved by Gansu Agricultural University.

### 2.2. Collection of Ovine Tissue and Blood Samples

The graphical scheme of the whole experiment is shown in Supplementary File 1. Four healthy 18-month-old Tibetan ewes were selected for the study. All these ewes were raised under the same environmental conditions in Gannan Tibetan Autonomous Prefecture (Gansu, China). When the ewes were slaughtered, seven tissue samples were collected for RNA isolation, including subcutaneous adipose, liver, lung, *longissimus dorsi* muscle, spleen, heart and kidney. The collected samples were frozen immediately in liquid nitrogen until RNA extraction.

Meanwhile, one of the four Tibetan ewes described above was selected, three pieces of subcutaneous adipose tissue samples about 1 cm^3^ were collected to culture ovine preadipocytes. Blood samples were also collected to extract genomic DNA for the amplification of the 3’-untranslated regions (3’UTR) sequence of *p27* and *KLF9* using EasyPure^®^ Blood genomic deoxyribonucleic acid Kit (TransGen Biotech, Beijing, China).

### 2.3. RNA Isolation and Reverse Transcription-Quantitative PCR (RT-qRCR) Analysis

The total RNA from ovine seven tissues was extracted using TRIzol (Vazyme, Nanjing, China), and the complementary DNA (cDNA) was synthesized using the HiScript III 1st Strand cDNA Synthesis Kit (Vazyme, Nanjing, China). *TBP* [20] and *U6* [21] were used as internal references for the normalization of mRNAs and miRNAs, respectively. The RT-qPCR analysis was carried out in triplicate using the 2× ChamQ SYBR qPCR Master system (Vazyme, Nanjing, China). The information of PCR primer for miRNAs and genes was listed in Table S1. The relative expression levels of the RNAs were calculated using a 2^−ΔΔCt^ method.

### 2.4. Isolation and Culture of Ovine Primary Preadipocytes

The ovine adipose tissue samples were purified by removing connective tissues and visible blood vessels, and then cut to 0.5–1.0 mm^3^ pieces. Then, 1.0 U/mL Dispase type II (Solarbio, Beijing, China) and 0.75 U/mL collagenase D (Solarbio, Beijing, China) were used to digest the piece of adipose tissue at 37 °C for 1 h. The ovine preadipocytes were isolated and collected through centrifugation. Subsequently, the cells were seeded into culture mask in triplicate and then cultured at 5% CO_2_ with 37 °C in growth medium, which was composed of DMEM-F/12 medium (Hyclone, Logan, UT, USA) and 10% fetal bovine serum (Invigentech, Xi’an, China). 

Based on the definition of preadipocyte growth [3], before the density of preadipocytes reaches 100%, the number of preadipocytes continuously increases and the process is referred to ‘proliferation’. When the density of preadipocytes reached 90% ~ 100%, the differentiation inducer was added into original growth medium to initiate the differentiation of preadipocytes. The differentiation inducer was composed of 1 μg/mL insulin, 0.1 μg/mL dexamethasone, and 27.8 μg/mL 3-isobutyl-1-methylxanthine. After 2 days of inducement, the preadipocytes were further differentiated in a maintenance medium (growth medium and 1 μg/mL insulin). After another 2 days, the maintenance medium was replaced with original growth medium until differentiation was completed. The differentiation process described above lasted for 8 days.

### 2.5. The Effect of miR-200b on Ovine Preadipocytes Proliferation

When the density of preadipocytes reached approximately 50%, miR-200b mimic, miR-200b inhibitor and each negative control (NC) were transfected into preadipocytes using INVI DNA RNA Transfection ReagentTM (Invigentech, Xi’an, China). The proliferation activity of preadipocytes was detected using a CCK-8 Cell Counting Kit (Vazyme, Nanjing, China) at 0, 6, 12, 24, and 48 h after transfection. The absorbance was measured at 450 nm wavelength using Varioskan LUX Multimode Reader (Thermo Fisher Scientific, Waltham, MA, USA). 

The expression levels of the target gene *p27* and proliferation marker genes cyclin dependent kinase 2 (*CDK2*), cyclin dependent kinase 4 (*CDK4*), CyclinB1 (*CCNB1*), CyclinD1 (*CCND1*), proliferating cell nuclear antigen (*PCNA*) and *p53* were detected in triplicate cells by RT-qPCR on day 2 after transfection.

The 5-ethynyl-2-deoxyuridine (EdU) assay was commonly used to detect the rate of DNA synthesis and then reflect the rate of cell proliferation [22,23]. For the EdU analysis, after transfection for 48 h, the final concentration of 10 μM EdU reagent A (Beyotime, Beijing, China) was supplemented in growth medium to culture ovine preadipocytes at 37 °C for 2 h. The preadipocytes were then fixed with 4% paraformaldehyde and further incubated with Click Reaction Solution (Beyotime, Beijing, China) for 30 min. The nucleus of ovine preadipocytes was stained with Hoechst 33342 for 30 min. Finally, the fluorescence of the stained nucleus was observed using an IX53 inverted fluorescence microscope (Olympus, Tokyo, Japan). Three images were randomly collected to statistically analyze the number of total preadipocytes and EdU-positive preadipocytes.

### 2.6. The Effect of miR-200b on the Differentiation of Ovine Preadipocytes

For investigating the expression profile of miR-200b in the differentiation process of ovine preadipocytes, RNA was extracted from adipocytes on day 0, 0.5, 1, 2, 4, 6, and 8 after the initiation of differentiation. The expression level of miR-200b was detected using RT-qPCR. Meanwhile, the expression level of adipogenesis marker gene peroxisome proliferator activated receptor gamma (*PPARγ*) was also detected.

When the density of preadipocytes reached approximately 90%, miR-200b mimic, miR-200b inhibitor and NC were also transfected into preadipocytes and the inducer described above was used to induce the differentiation of preadipocytes. On day 8 after transfection (when the differentiation of ovine preadipocytes finished), the expression levels of the target gene *KLF9*, and differentiation marker genes fatty acid binding protein 4 (*aP2*), *PPARγ*, fatty acid synthase (*FASN*), solute carrier family 2 member 4 (*GLUT4*), lipoprotein lipase (*LPL*) and CCAAT/enhancer binding protein beta (*C/EBPβ*) were detected using RT-qPCR.

For Oil Red O staining, mature adipocytes on day 8 of induced differentiation were fixed using 4% paraformaldehyde solution for 30 min, and then stained with 1% Oil Red O solution (Solarbio, Beijing, China) for 20 min. The stained lipid droplets in the cytoplasm of adipocytes were observed using an IX53 inverted fluorescence microscope (Olympus, Tokyo, Japan). Three images were randomly collected for each observation field to calculate the area of Oil Red O staining.

### 2.7. The Prediction of the Target Genes of miR-200b

TargetScan (v7.2) and miRDB (v4.0) were used to predict the target genes of miR-200b, and a total of 626 target genes were obtained. Based on their roles in adipogenesis [24,25,26], *p27* and *KLF9* were selected to perform dual luciferase reporter assay for testing their target relationship with miR-200b.

### 2.8. Dual Luciferase Reporter Assay

PCR primers for amplifying the 3′UTR sequence of *p27* and *KLF9* were designed (Table S1). Amplified 3′UTR sequence was ligated into the 3’-end of the Ranilla luciferase reporter gene of the pmiR-RB-Report™ vector (RiboBio, Guangzhou, China) to construct a wild-type 3′UTR dual luciferase reporter vector. The miR-200b seed binding sequence inserted in the wild-type 3′UTR reporter vectors was mutated to construct a mutate-type 3′UTR dual luciferase reporter vector using a Mut Express II Fast Mutagenesis Kit (Vazyme, Nanjing, China). 

In total, 500 ng of wild-type or mutant-type dual luciferase reporter vector and 100 pmoL of miR-200b mimic or NC were co-transfected into HEK293 cells. The Dual-luciferase Reporter^®^ Assay System (Promega, Madison, WI, USA) was used to detect the luciferase activity of HEK293 cells 48 h after transfection.

### 2.9. The Effect of p27 and KLF9 on Ovine Preadipocytes Proliferation and Differentiation

We investigated both the effect of *p27* on the proliferation of ovine preadipocytes, and the effect of *KLF9* on the differentiation of adipocytes.

The small interference RNA (siRNA) of the miR-200b predicted target genes *p27* and *KLF9* (named si-*p27* and si-*KLF9*) was synthesized by GenePharma Ltd. (Shanghai, China). The PCR primers (Table S1) were designed to amplify the coding sequence (CDS) region of *p27* and *KLF9* using cDNA from ovine subcutaneous adipose tissue. The CDS of *p27* obtained was ligated into pcDNA3.1(+) eukaryotic expression plasmid (Invitrogen, California, USA) to construct an expression vector (named pcDNA3.1- *p27*). The same method was used to construct the expression vector of *KLF9* (named pcDNA3.1- *KLF9*).

When the density of preadipocytes reached 50%, 200 pmoL of si-*p27* and 2 μg of pcDNA3.1-*p27* were transfected into ovine preadipocytes using INVI DNA RNA Transfection ReagentTM (Invigentech, Xi’an, China), respectively. On day 2 after transfection, the EdU staining of preadipocytes was performed and the expression levels of *CDK2*, *CDK4*, *CCND1* and *PCNA* were also detected.

When the density of preadipocytes reached approximately 90%, si-*KLF9* and pcDNA3.1-*KLF9* were transfected into preadipocytes, and the inducer described above was used for the differentiation of preadipocytes. On day 8 after transfection (when the differentiation of ovine preadipocytes finished), the Oil Red O staining areas of adipocytes and the expression levels of *aP2*, *PPARγ*, *LPL* and *GLUT4* were detected.

### 2.10. Statistical Analysis

The differences between groups were analyzed using two-tailed student’s *t*-test in SPSS 22.0 (IBM, NY, USA). Data are presented as means ± SD. All *p*-values were considered statistically significant when *p* < 0.05.

## 3. Results

### 3.1. The Expression Profile of miR-200b

**Figure 1:**
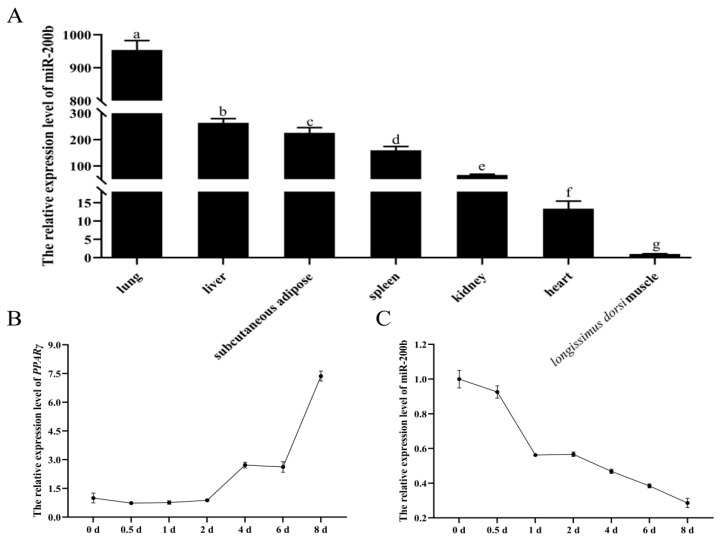
The expression profiles of miR-200b and *PPARγ*. (**A**) The expression level of miR-200b in the ovine seven different tissues. The expression levels of *PPARγ* (**B**) and miR-200b (**C**) during ovine preadipocyte differentiation. The values are shown as mean ± SD (*n* = 4). Values with different lowercase letters are different at *p* < 0.05.

The RT-qPCR analysis indicated that miR-200b was highly expressed in lung, liver, subcutaneous adipose and spleen. It had a lower expression in kidney, heart and *longissimus dorsi* muscle (Figure 1A).

*PPARγ* is a marker gene of adipogenesis, and its expression level is positively correlated with the degree of differentiation in preadipocytes. When the induction of preadipocytes differentiation initiated, the expression of *PPARγ* significantly increased on day 2 after differentiation and achieved a peak level on day 8 (Figure 1B). This suggests that the adipogenic differentiation of preadipocytes was normal. However, the expression of miR-200b continuously decreased from the initiation of preadipocytes differentiation (Figure 1C).

### 3.2. MiR-200b Promotes Ovine Preadipocytes Proliferation

**Figure 2:**
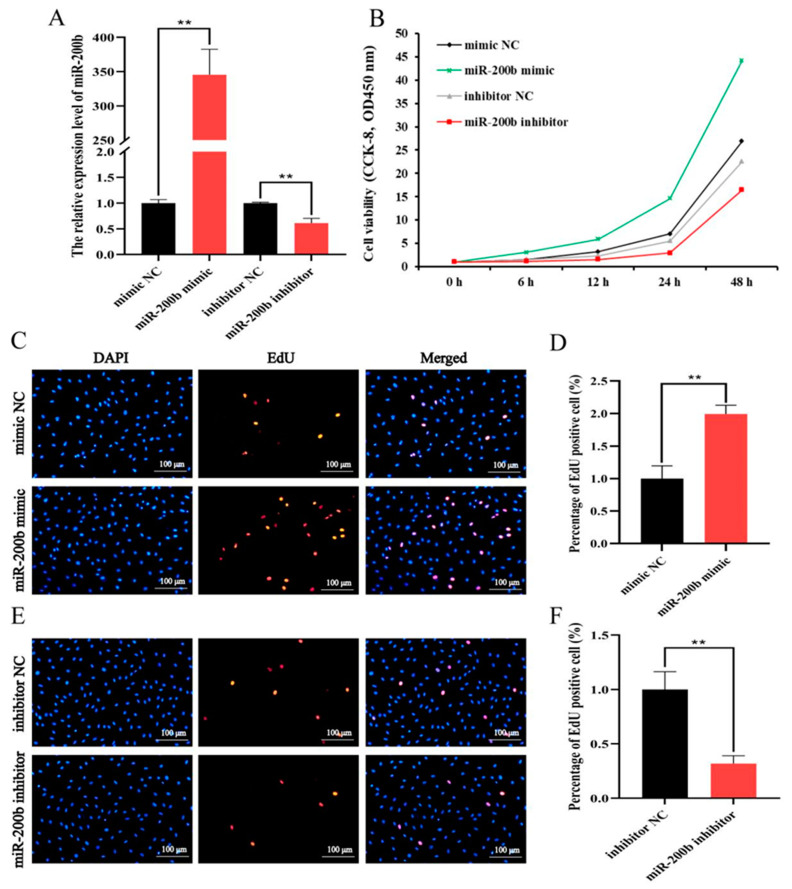
The effects of miR-200b on the proliferation of ovine preadipocytes transfected with miR-200b mimic, miR-200b inhibitor and negative control (NC). (**A**) The transfection efficiency of miR-200b in ovine preadipocytes on day 2 after transfection. (**B**) The proliferation activity of ovine preadipocytes was analyzed using CCK-8. (**C**,**E**) The EdU analysis of ovine preadipocytes. The images of DAPI and EdU reflect the total number of preadipocytes and the number of EdU-labeled preadipocytes, respectively. The image of Merged reflects the proportion of EdU-labeled preadipocytes in the total preadipocytes. (**D**,**F**) The proportion of EdU-labeled preadipocytes in the total preadipocytes. The values represent mean ± SD (*n* = 3), ** *p* < 0.01.

The miR-200b mimic significantly increased the expression level of miR-200b in ovine preadipocytes compared to miR-200b NC. On the contrary, miR-200b inhibitor decreased the expression level of miR-200b (Figure 2A). These results suggest that miR-200b mimic and miR-200b inhibitor were successfully transfected into ovine preadipocytes. The proliferation activity assay results showed the miR-200b mimic increased the viability of ovine preadipocytes, while miR-200b inhibitor decreased the viability (Figure 2B).

The EdU staining result indicated that miR-200b mimic significantly increased the number of EdU-labeled preadipocytes (Figure 2C,D), whereas miR-200b inhibitor exhibited the opposite effect with miR-200b mimic (Figure 2E,F).

**Figure 3:**
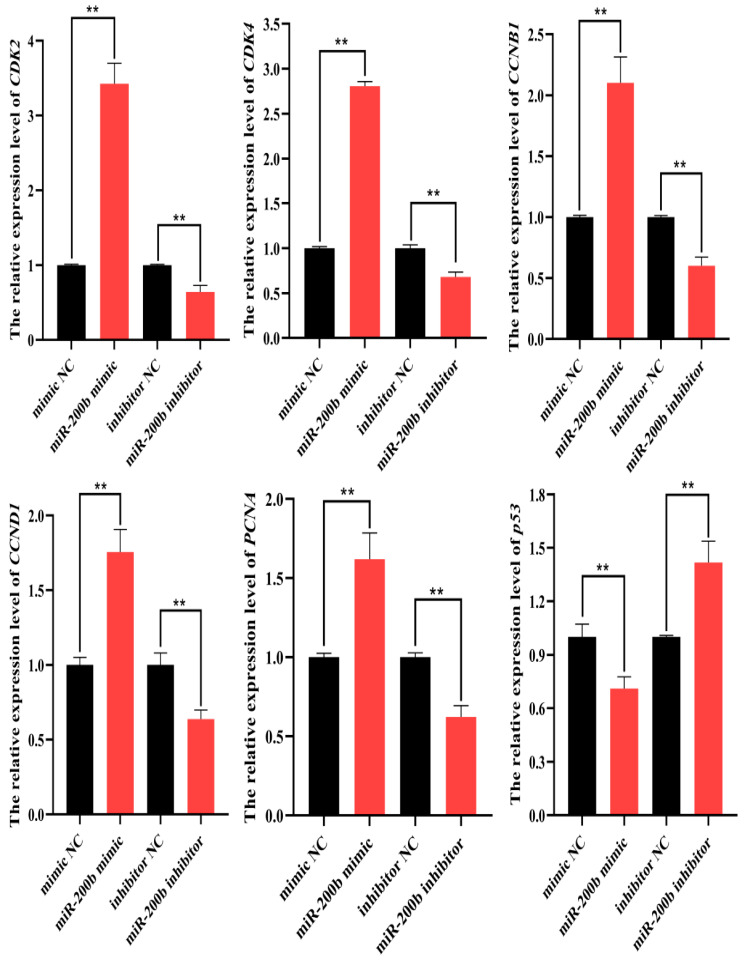
The expression levels of *CDK2*, *CDK4*, *CCNB1*, *CCND1*, *PCNA* and *p53* in ovine preadipocytes on day 2 after the transfection with miR-200b mimic, miR-200b inhibitor and NC. The values represent mean ± SD (*n* = 3), ** *p* < 0.01.

The RT-qPCR results showed that the expression levels of proliferation marker genes *CDK2*, *CDK4*, *CCNB1*, *CCND1* and *PCNA* were upregulated, while *p53* was downregulated in the preadipocytes transfected with miR-200b mimic when compared to NC group. On the contrary, miR-200b inhibitor decreased the expression of *CDK2*, *CDK4*, *CCNB1*, *CCND1* and *PCNA*, but increased the expression of *p53* (Figure 3).

### 3.3. The miR-200b Negatively Regulates Ovine Adipocyte Differentiation

**Figure 4:**
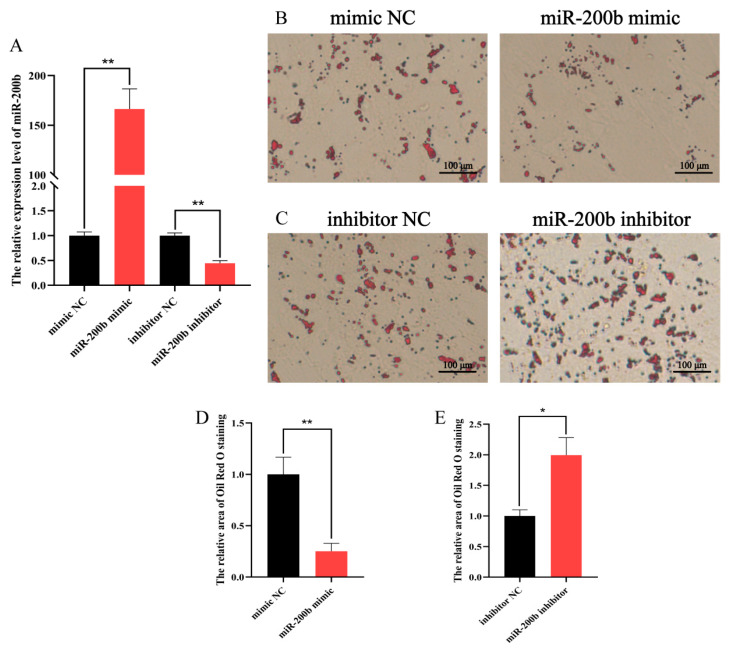
The effect of miR-200b on ovine adipocyte differentiation on day 8 after transfection with miR-200b mimic, miR-200b inhibitor and negative control (NC). (**A**) The transfection efficiency of miR-200b. (**B**,**C**) The Oil Red O staining results of ovine adipocytes. (**D**,**E**) The area of Oil Red O staining counted by the ImageJ software. The values represent mean ± SD (*n* = 3), * *p* < 0.05, ** *p* < 0.01.

The results presented in Figure 4A showed that miR-200b mimic, miR-200b inhibitor, and NC were efficiently transfected into ovine adipocytes on day 8. The Oil Red O staining results showed that miR-200b mimic significantly decreased the accumulation of lipid droplets when compared to its NC group in adipocytes (Figure 4B). On the contrary, miR-200b inhibitor significantly increased the number of lipid droplets (Figure 4C). The area of Oil Red O counted further confirmed these, namely, miR-200b mimic decreased the area of Oil Red O staining by 75% when compared to mimic NC (Figure 4D). In contrast, miR-200b inhibitor increased the area of Oil Red O staining by 99% (Figure 4E).

**Figure 5:**
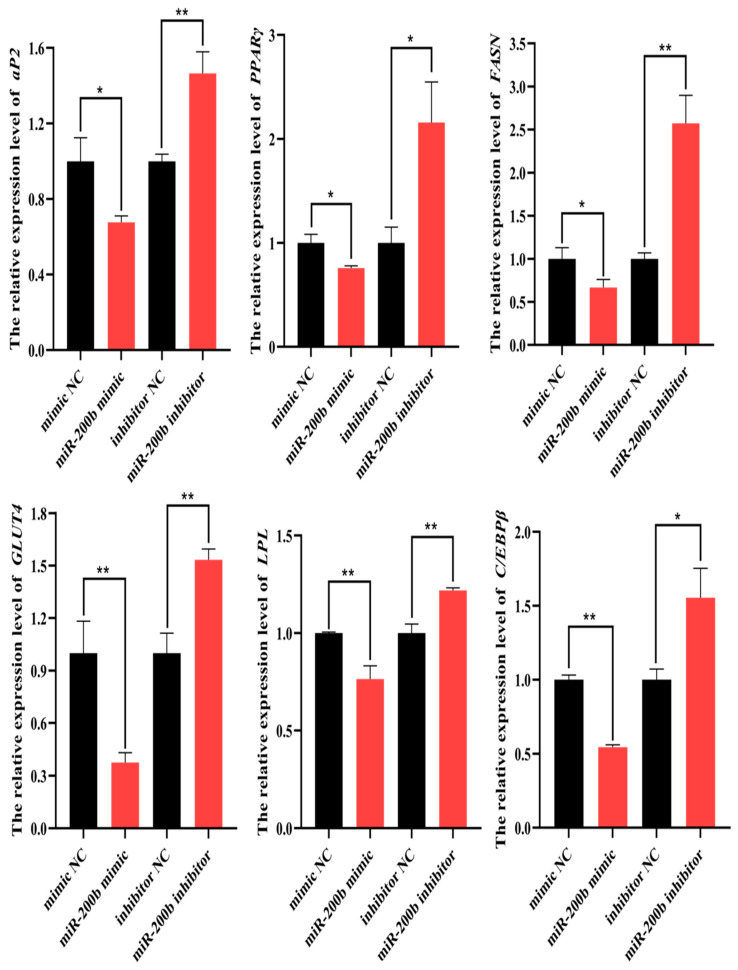
The expression levels of *aP2*, *PPARγ*, *FASN*, *GLUT4*, *LPL* and *C/EBPβ* in ovine adipocytes on day 8 after transfection with miR-200b mimic, miR-200b inhibitor and NC. The values represent mean ± SD (*n* = 3), * *p* < 0.05, ** *p* < 0.01.

On day 8 after transfection with miR-200b mimic, the expression levels of lipogenesis marker genes *aP2, PPARγ*, *FASN*, *GLUT4*, *LPL* and *C/EBPβ* significantly decreased in ovine adipocytes when compared to NC group. On contrary, the expression levels of the genes were elevated when miR-200b was silenced in ovine adipocytes using miR-200b inhibitor (Figure 5).

### 3.4. MiR-200b Targets the 3′UTR Region of p27 and KLF9

**Figure 6:**
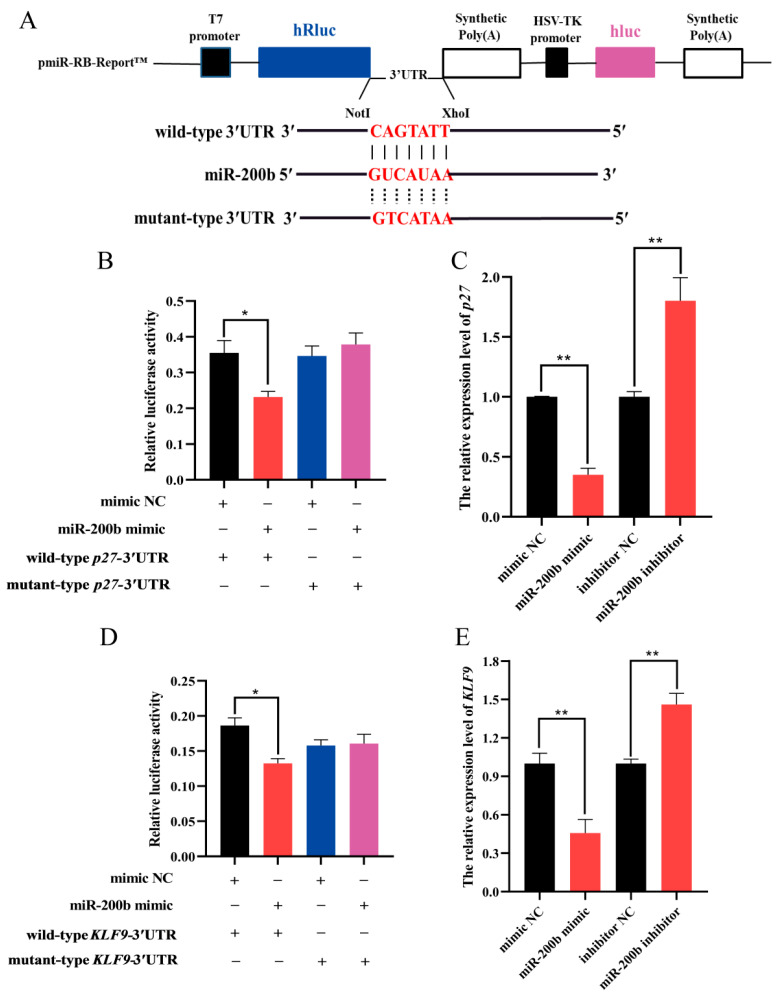
The miR-200b targets *p27* and *KLF9* in ovine preadipocytes. (**A**) The structural diagram of wild-type and mutant-type dual luciferase reporter vectors. (**B**,**D**) The luciferase activity was analyzed when miR-200b mimic, NC and wild-type or mutant-type dual luciferase reporter vectors were co-transfected into HEK293 cells. (**C**) The expression level of *p27* was detected in preadipocytes on day 2 after the transfection of miR-200b mimic, miR-200b inhibitor, and NC. (**E**) The expression level of *KLF9* in adipocytes on day 8 after transfecting with miR-200b mimic, miR-200b inhibitor, and NC. The values represent mean ± SD (*n* = 3), * *p* < 0.05, ** *p* < 0.01.

The wild-type and mutant-type dual luciferase reporter vectors for *p27*-3′UTR and *KLF9*-3′UTR were structured (Figure 6A). The dual luciferase reporter assay showed that miR-200b mimic significantly decreased the luciferase activity of *p27* and *KLF9* in HEK293 cells transfected with wild-type dual luciferase reporter vectors when compared to NC group (Figure 6B,D). However, in HEK293 cells transfected with mutant-type dual luciferase reporter vectors, miR-200b had no significant effect on the luciferase activity of *p27* or *KLF9* (Figure 6B,D). These results suggest that *p27* and *KLF9* are target genes of miR-200b.

The RT-qPCR results showed that miR-200b mimic significantly decreased the expression levels of *p27* and *KLF9* in preadipocytes, while the expression of the two target genes was remarkably increased in ovine preadipocytes transfected with miR-200b inhibitor (Figure 6C,E).

### 3.5. p27 Inhibits Proliferation of Ovine Preadipocytes

**Figure 7:**
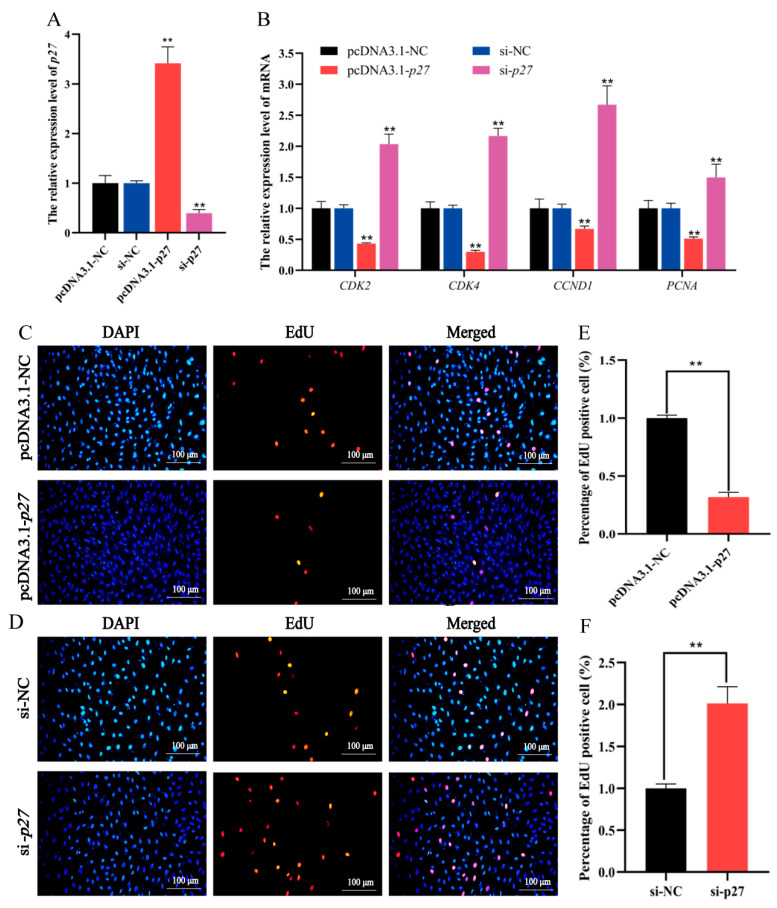
The effect of *p27* on ovine preadipocyte proliferation when pcDNA3.1-*p27*, si-*p27* and their NC groups were transfected into preadipocytes. (**A**) The expression level of *p27*. (**B**) The expression levels of *CDK2*, *CDK4*, *CCND1* and *PCNA*. (**C**,**D**) The EdU staining result. (**E**,**F**) The percentage of EdU positive preadipocytes counted by the ImageJ software. The values represent mean ± SD (*n* = 3), ** *p* < 0.01.

The RT-qPCR results showed that the transfection of pcDNA3.1-*p27* significantly enhanced *p27* expression level, whereas the transfection of si-*p27* significantly inhibited the expression level of *p27* in preadipocytes (Figure 7A). This suggests that pcDNA3.1-*p27* and si-*p27* were successfully transfected into preadipocytes.

In ovine preadipocytes transfected into pcDNA3.1-*p27*, the expression levels of proliferation marker genes *CDK2*, *CDK4*, *CCND1* and *PCNA* significantly reduced (Figure 7B), and the number of EdU-labeled preadipocytes also decreased (Figure 7C,E). The opposite result with pcDNA3.1-*p27* was observed in preadipocytes transfected with si-*p27* (Figure 7B,D,F).

### 3.6. KLF9 Promotes Differentiation of Ovine Adipocytes

**Figure 8:**
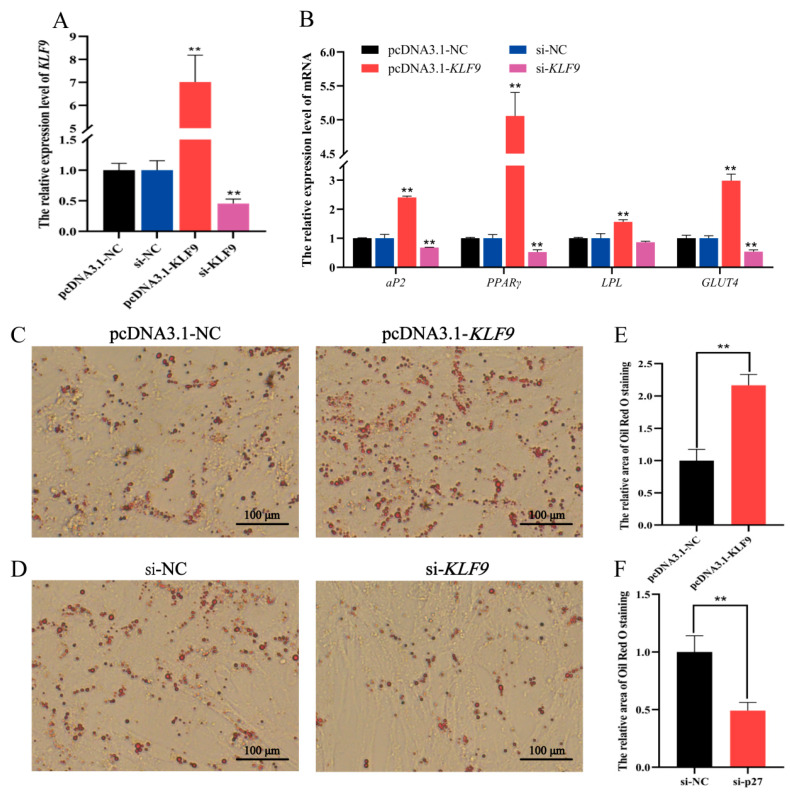
The effect of *KLF9* on ovine adipocyte differentiation when pcDNA3.1-*KLF9*, si-*KLF9* and their NC were transfected into differentiated preadipocytes. (**A**) The expression level of *KLF9*. (**B**) The expression levels of *aP2*, *PPARγ*, *LPL* and *GLUT4*. (**C**,**D**) The Oil Red O staining results. (**E**,**F**) The relative area of Oil Red O staining was counted using the ImageJ software. The values represent mean ± SD (*n* = 3), ** *p* < 0.01.

Figure 8A shows that pcDNA3.1-*KLF9* and si-*KLF9* were efficiently transfected into adipocytes. When compared to pcDNA3.1-NC group, the transfection of pcDNA3.1-*KLF9* significantly enhanced the expression levels of adipogenesis marker genes *aP2*, *PPARγ*, *LPL* and *GLUT4* in adipocytes (Figure 8B) and the accumulation of lipid droplets (Figure 8C,E). However, si-*KLF9* decreased the expression levels of the adipogenesis marker genes (Figure 8B) and the area of Oil Red O staining (Figure 8D,F).

## 4. Discussion

Over that last decade, miRNAs have been shown to be essential molecules in the regulation of the proliferation and adipogenesis of preadipocytes in domestic animals [21,27]. However, this is the first study to systematically report the expression profile of miR-200b, and its effect on the proliferation and differentiation of ovine preadipocytes.

In this study, miR-200b exhibited higher expression levels in lung, liver, subcutaneous adipose and spleen tissues than in kidney, heart and *longissimus dorsi* muscle. This was consistent with the observation in mice, in which miR-200b has high expression level in liver and adipose tissue [19]. These results indicate that the higher expression of miR-200b in liver and adipose tissue may be related to its roles in lipid accumulation and metabolism. Meanwhile, the expression level of miR-200b continuously decreased with the differentiation and maturation of ovine preadipocytes. The expression tendency of miR-200b was opposite with *PPARγ*, which is a marker gene of adipogenic differentiation, and its expression level is positively correlated with the degree of differentiation of preadipocytes [28]. Shen et al. also found that the expression of miR-200b was continuously reduced in differentiated mouse 3T3-L1 preadipocytes [17]. This suggests that miR-200b may negatively regulate the differentiation and lipid accumulation of ovine preadipocytes. The subsequent Oil Red O staining and RT-qPCR experiments also demonstrated this when miR-200b was over-expressed or inhibited in adipocytes.

In the study, the over-expressed miR-200b increased the viability of ovine preadipocytes, the number of EdU-labeled preadipocytes and the expression levels of proliferation marker genes *CDK2*, *CDK4*, *CCNB1*, *CCND1* and *PCNA*, but inhibited *p53* expression. This result is in accordance with another observation that miR-200b mimic dramatically accelerated the proliferation vitality of 3T3-L1 preadipocytes [17]. *CDK2*, *CDK4*, *CCNB1* and *CCND1* can accelerate the cell cycle moving from G1 to S phase and then promote the proliferation of various cell types including mouse embryonic fibroblasts [29], mouse neural progenitor cells [30] and human hepatocellular carcinoma [31]. *p53* has been found to negatively regulate the proliferation of human colon cancer cells by inhibiting the activity of CDK1 [32]. These results indicate that miR-200b promotes the proliferation of ovine preadipocytes. The positive effect of miR-200b on the proliferation has been reported in many types of cells. For example, miR-200b has been found to promote the proliferation of human hepatic stellate cells via downregulating *FOG2* expression [33]. The same as miR-200b, miR-429 is also a member of the miR-200b/c/429 family and shares the same conservative seed sites and target genes with miR-200b [34]. The miR-429 has been confirmed to promote the proliferation of porcine preadipocytes [25].

In differentiated ovine preadipocytes investigated in the study, the over-expressed miR-200b decreased the accumulation of lipid droplets and the expression of *aP2*, *PPARγ*, *FASN*, *LPL*, *GLUT4* and *C/EBPβ*. These results are in accordance with the finding that miR-200b mimic inhibited lipid accumulation and differentiation of 3T3-L1 preadipocytes [17]. The expression levels of *aP2*, *PPARγ*, *FASN*, *LPL*, *GLUT4* and *C/EBPβ* were positively correlated with the degree of the differentiation of adipocytes and are believed to be lipogenesis marker genes. For example, the upregulation of *PPARγ* expression induced lipid transport and triglyceride synthesis in adipocytes, and ultimately resulted in the hypertrophy of adipose tissue [28]. *C/EBPβ* is an essential transcription factor for initiating adipocytes differentiation. The over-expressed *C/EBPβ* was sufficient to launch the differentiation of mouse 3T3-L1 cells without normally required hormonal inducers, while knockout of *C/EBPβ* impaired development of adipose tissue in mice [35,36]. Our results therefore suggest that miR-200b inhibits the differentiation of ovine preadipocytes.

It was verified in the study that miR-200b targets *p27* and *KLF9*, and as a result inhibits the expression levels of *p27* and *KLF9* in ovine preadipocytes. The target relationship between miR-200b and *p27* has been reported in other types of cells. For example, miR-200b significantly decreased the relative fluorescence activity of wild-type *p27*-3′UTR reporter vector and downregulated the expression of *p27* in both human Tenon’s capsule fibroblast cells [37] and colorectal cancer cells [26]. However, for miR-200b/c/429 family members, the target relationship of *KLF9* was only reported with miR-200c, but not miR-200b. Briefly, *KLF9* has been reported to be a target gene of miR-200c in human endometrial cancer cells [24] and porcine adipocytes [25]. However, in this study, we found that *KLF9* was also a target gene of miR-200b. This is not surprising though, as miR-200b and miR-200c have the same conservative seed sequences [34].

The over-expressed *p27* decreased the expression levels of proliferation markers genes *CDK2*, *CDK4*, *CCND1* and *PCNA*, and the number of EdU-labeled preadipocytes. Together, our results suggest that *p27* inhibits the proliferation of ovine preadipocytes. The inhibitory effect of *p27* on cell proliferation has been reported. Higher proliferation activity was observed in astrocytes of mice with the knockout of *p27* when compared to wild-type mice [38]. Another study also found that *p27* knock-out mice had a 1.7-fold higher adipocyte number than wild-type mice [39]. Given the target relationship between miR-200b and *p27*, together with the effect of miR-200b and *p27* on the proliferation of ovine preadipocytes, it was concluded in the study that miR-200b promotes the proliferation of ovine preadipocytes by inhibiting the expression of *p27*.

It was found in the study that *KLF9* promotes the differentiation of ovine adipocytes. The positive effect of *KLF9* on adipogenesis has also been reported. Pei et al. found that KLF9 trans-activated the transcription of *PPARγ* to initiate the differentiation of mouse 3T3-L1 cells [40]. On the contrary, the differentiation of 3T3-L1 cells was suppressed when *KLF9* was knocked out. Based on the results described above, it was also inferred that miR-200b suppresses the differentiation of ovine preadipocytes by inhibiting *KLF9* expression.

## 5. Conclusions

In summary, miR-200b promoted the proliferation of ovine preadipocytes via targeting *p27*, while it also suppressed the differentiation of ovine adipocytes through targeting *KLF9*. This study contributes to understanding the roles of miR-200b in the proliferation and differentiation of ovine preadipocytes, and it also provides a theoretical platform for improving fat content by manipulating the expression of miR-200b in sheep.

## Data Availability

All relevant data is given in the paper. Additional information can be requested from the corresponding authors upon reasonable request.

## Supplementary Materials

The following are available online at https://www.mdpi.com/article/10.3390/ani11082417/s1, Table S1: List of the PCR primers used in this study; File S1: The graphical scheme of whole experiment.
