# Supplementary material for: MicroRNA-200b Regulates the Proliferation and Differentiation of Ovine Preadipocytes by Targeting p27 and KLF9
Source: Animals (Basel). 2021 Aug 17;11(8):2417. doi: 10.3390/ani11082417 (PMC8388755; doi:10.3390/ani11082417)
Supplement: Supplementary file 1 [file animals-11-02417-s001.zip › Table S1.pdf]

Table S1. List of the PCR primers used in this study

Gene	Forward primer sequence (5'–3')	Reverse primer sequence (5'–3')	Reference sequence	Designed purpose
aP2	GTCCTTCAAATTGGGCCAGG	ACTCTGGTAGCAGTGACACC	NM_001114667.1	RT-qPCR
PPARγ	GAGCCTTCCAACTCCCTCAT	ATGAGACATCCCCACAGCAA	NM_001100921.1	RT-qPCR
C/EBPβ	CATCGACTTCAGCCCCTACC	CCGCCTTCTTGCAGTTCTTG	XM_004014883.4	RT-qPCR
LPL	AGCTGCAGAAAGAACCGTTG	GAGATCTCGAAGGCCTGGTT	NM_001009394.1	RT-qPCR
FASN	ACACAAATTGAGCAGCCCTG	TGCCGCTCTTGTACACTGTA	XM_027974304.1	RT-qPCR
GLUT4	TGGCTACAACATTGGGGTCA	ATCATGCCACCCACAGAGAA	XM_027974995.1	RT-qPCR
KLF9	CTGAACAAGTACCGACCCAT	ATAGACTTTCCCACAGCCAC	XM_004004321.4	RT-qPCR
CDK2	GACCAGCTCTTCCGGATCTT	ACAAGCTCCGTCCATCTTCA	NM_001142509.1	RT-qPCR
CDK4	ACTTTGTGGCCCTCAAGAGT	CCTGAGGTCTTGGTCCACAT	NM_001127269.1	RT-qPCR
CCNB1	CCCTCCAGAAATCGGTGACT	AGCTCAACATCAACCTCTCCA	XM_027980034.1	RT-qPCR
CCND1	CATCGAGCACTTCCTCTCCA	CGGGTCACATCTGATCACCT	XM_027959928.1	RT-qPCR
PCNA	TCAAGTGGCGTGAACCTACA	TACTAGTGCCAAGGTGTCCG	XM_004014340.4	RT-qPCR
p53	CGGCTTGCAGAAACCTCTTT	CCCTTTTCTACCTCCTGCCA	XM_004006850.4	RT-qPCR
p27	CGGCTTGCAGAAACCTCTTT	CCCTTTTCTACCTCCTGCCA	XM_004006850.4	RT-qPCR
TBP	ACAGCCTCCCACCATATGCCC	GCTGTGGAGTCAGTCCTGTGC	XM_027972700.1	RT-qPCR
U6	TGGAACGCTTCACGAATTTGCG	GGAACGATACAGAGAAGATTA	XM_012096728.2	RT-qPCR
KLF9 3'UTR-wild	CCGCTCGAGAGACAATCTTTATAGTTTCAGG	AAATATGCGGCCGCGTACAAAATACCTACTGTCAGAA	XM_004004321.4	Amplification of 3' UTR of *KLF9*
KLF9 3'UTR-Mut	GTAGTCATAAAGTGACCTAAGTTATTTTGCTGTCCC	GGTCACTTTATGACTACAAAAATAAAACTGATTAAACAGTTG	XM_004004321.4	Construction of mutant 3' UTR
KLF9 CDS	GGGGTACCATGTCCGCAGCCGCCTAC	GCTCTAGATCACAAAGGGCTGGCCAG	XM_004004321.4	Amplification of CDS sequence

p27 3'UTR-wild	CCGCTCGAGCGCTGACTCCAAGAACGG	AAATATGCGGCCGCTTGGCTCAGTATGCAACCTTT	XM_004006850.4	Amplification of 3' UTR of *p27*
p27 3'UTR-Mut	CCGTCATAACATCAGTCTGGTAATCACTCCA	GACTGATGTTATGACGGTAAATTCCAATTGTAAGGGAGAC	XM_004006850.4	Construction of mutant 3' UTR
p27 CDS	GGGGTACCATGTCAAACGTGCGAGTGTCTA	GCTCTAGATTACGTCTGACGCCTTCTGAGG	XM_004006850.4	Amplification of CDS sequence
