# Supplementary figures and images for: MicroRNA-200b Regulates the Proliferation and Differentiation of Ovine Preadipocytes by Targeting p27 and KLF9
Source: Animals (Basel). 2021 Aug 17;11(8):2417. doi: 10.3390/ani11082417 (PMC8388755; doi:10.3390/ani11082417)

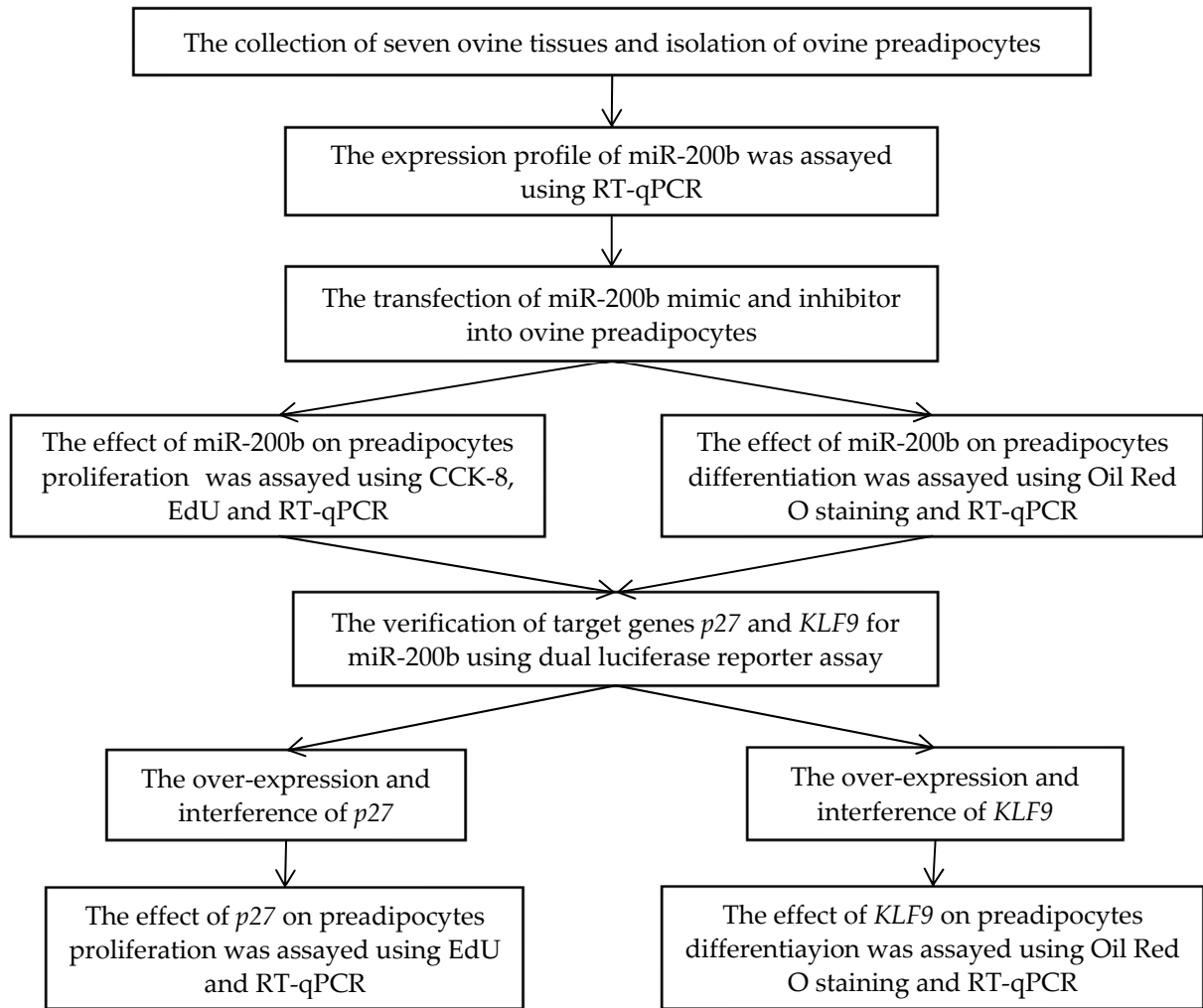

**File S1.** The graphical scheme of whole experiment

Supplement: Supplementary file 1 [file animals-11-02417-s001.zip › Supplementary File 1.pdf]
